# Performance of UF-5000 in rapidly screening out urinary tract infection, predicting Gram-negative bacteria infection

**DOI:** 10.1128/spectrum.00301-24

**Published:** 2024-11-11

**Authors:** Wei Lu, Zhaoxing Li, Tong Zhang, Yan Li, Huange Zhu, Ya Zhao, Ke Lei, Zhi Guo, Jing Zhang, Juan Guo, Lei Zhang

**Affiliations:** 1 Department of Laboratory, the Second Affiliated Hospital of Xi'an Jiao Tong University, Xi’an, China; Beijing Institute of Genomics, Beijing, China

**Keywords:** urinary tract infections, UF-5000, urine analysis, urine culture, B_FSC, B_FLH

## Abstract

Urine culture is the gold standard for identifying microorganisms in urine. However, the process is time-consuming and usually takes days to get the results, which impedes timely treatment. This study aimed to evaluate the performance of UF-5000 in excluding bacterial urinary tract infection (UTI) and detecting the presence of Gram-negative bacteria. A total of 1,522 urine specimens were subjected to routine urine analysis and culture. Bacteria, white blood cell counts, and UF-5000 bacteria information flags were compared with urine culture. Bacteria forward scatter (B_FSC), and bacteria fluorescent light intensity (B_FLH) parameters were assessed to determine the optimal angle for discriminating bacterial growth patterns, which was verified in the validation cohort. The optimal cut-off values were 42.2/µL and 100.2/µL to rule out UTI for bacteria at ≥10^4^ CFU/mL and ≥10^5^ CFU/mL, respectively. The agreement of UF-5000 bacterial classification and urine culture was fair (Kappa = 0.227). Regarding the discrimination of bacterial groups, “Gram Neg?” was associated with a specificity of 94.0% and a positive predictive value of 87.2% in the detection of Gram-negative bacteria. By estimating the B_FSC and B_FLH parameters, the best angle was 28° for distinguishing Gram-negative and Gram-positive bacteria. Among the 136 urine specimens with the low angle pattern (<28°) in the validation cohort, 127 specimens were confirmed to contain Gram-negative bacteria. The UF-5000 analyzer is a suitable and rapid tool to exclude negative urine specimens, and bacterial information flags for Gram-negative bacteria could be trusted by clinicians.

**IMPORTANCE:**

The strength of our study relied on being the first study assessing the bacteria forward scatter (B_FSC) and bacteria fluorescent light intensity (B_FLH) research parameters with a UF-5000 urine analyzer and establishing the best angle for distinguishing Gram-negative and Gram-positive bacteria. When the bacterial scatter plot angle is less than 28°, the possibility of Gram-negative bacterial infection is more than 80%. Meanwhile, we find that UF-5000 bacterial information flags have a significant advantage in detecting Gram-negative bacteria with a specificity of over 90% and a positive predictive value of over 80%.

## INTRODUCTION

Urinary tract infections (UTIs) are an inflammatory response to pathogenic microorganisms in the urinary tract and are common in both inpatients and outpatients (1, 2). Generally, women and the elderly are at a higher risk (3). UTIs are diagnosed based on clinical symptoms and positive urine cultures. Urine culture is the gold standard for identifying microorganisms in urine. However, the process is time-consuming and usually takes 1–2 days to obtain the results. Moreover, most culture specimens are negative. Some patients are given empiric medication before urine culture results are available, which leads to antibiotic resistance. Therefore, a fast and reliable method is needed to exclude negative specimens.

Previous meta-analysis studies showed that the bacteria (BACT) and white blood cell (WBC) counts of urine flow cytometer might be used as parameters for UTI screening (4, 5). Studies have indicated that UF-1000i has a limited ability to discriminate between Gram-negative bacteria and Gram-positive bacteria (6, 7). Recently, a new generation of urine flow cytometers, UF-5000 (Sysmex Corporation, Kobe, Japan), was launched. The urine sediment analyzers’ work is based on fluorescence flow cytometry and urine particles are classiﬁed and counted according to their size, shape, volume, and staining features. In addition, the bacteria in a sample can be flagged as Gram-negative, Gram-positive, or mixed [“Gram Negative?” (GN), “Gram Positive?” (GP), “Gram Pos/Neg?” (GP/GN)] when the thresholds for the WBC count (≥10/µL) and bacterial count (≥100/µL) are met. Specimens are flagged as “unclassified” when bacterial classification is not possible. Furthermore, bacterial forward scatter (B_FSC) and fluorescent light intensity (B_FLH) can be assessed to the bacterial morphological information. Many previous studies have compared the results of urine sediment analyzers with culture results (8–13); however, few studies have evaluated the ability of UF-5000 to discriminate bacterial growth patterns (14–16), and no study has assessed the B_FSC and B_FLH parameters of UF-5000 to discriminate between Gram-negative and Gram-positive bacteria. Previous research had reported that the UF-1000i could preliminarily identify most Gram-negative bacteria based on the B_FSC and B_FLH parameters (17, 18). Therefore, we wanted to explore the performance of FSC and FLH parameters in identifying bacteria patterns with a UF-5000 urine analyzer. Negative results could be reported within minutes and positive urine samples that needed to be cultured could be immediately assessed by looking at the value of the B_FSC and B_FLH parameters. This morphological information could establish a more specific antimicrobial treatment in real-time while awaiting results for sensitivity testing.

Our study aimed to establish the best BACT and WBC cut-off values to exclude negative urine culture specimens. Additionally, we evaluated the accuracy of the Sysmex UF-5000 in discriminating bacterial growth patterns. Moreover, the optimal angle for distinguishing Gram-negative and Gram-positive bacteria was established and validated.

## MATERIALS AND METHODS

### Urine specimens collection

We conducted a prospective study in the Second Affiliated Hospital of Xi’an Jiao Tong University from 11 May to 12 August 2022. A total of 1,522 urine specimens were divided into a training cohort (*n* = 823) and a validation cohort (*n* = 699). Among the 823 urine specimens from 642 patients, 267 (41.6%) were male, and 375 (58.4%) were female. Their median age was 60 (51–72) years. Hospitalized patients (*n* = 619, 96.5%) accounted for the majority of the patients.

Urine specimens were collected in sterile containers without preservatives and delivered to the microbiology laboratory shortly after sample collection. The urine specimens were inoculated first, and the remaining specimens were used for routine urinalysis. If urine specimens were not processed immediately, they were stored at 2°C–8°C. All specimens should be analyzed within 4 hours. If these conditions were not met, specimens were excluded from our cohort. This study was performed in compliance with good clinical practice, and the trial protocol was approved by the Ethics Committee.

### Sysmex UF-5000

All urine specimens were analyzed with the Sysmex UF-5000 instrument according to the manufacturer’s instructions. The following parameters were collected: bacterial count (BACT × 10^6^/L), white blood cell count (WBC × 10^6^/L), yeast-like cell count (YLC × 10^6^/L), and epithelial cell count (EC × 10^6^/L). Moreover, we assessed the scatter diagrams and histograms of the bacterial distribution. B_FSC and B_FLH parameters were gathered from the UF-5000 sediment analyzers. Before testing the urine specimens, the devices were confirmed to have met the routine quality control and to pass regular calibration and performance verification (including precision, carryover rate, linear range, and reportable range validations).

### Urine culture

Well-mixed urine samples were inoculated and streaked onto blood agar and MacConkey agar plates (Autobio, Zhengzhou, China). After 18–24 hours of aerobic incubation at 37°C, the bacterial load was counted and expressed as colony-forming units per milliliter (CFU/mL).

In our study, positive urine culture results were defined as follows: (i) growth of bacteria ≥10^4^ CFU/mL and (ii) growth of bacteria ≥10^5^ CFU/mL. If bacterial counts were ≥10^4^ CFU/mL, bacterial identification and antimicrobial susceptibility analysis were performed using a VITEK2-compact automated system (Marcy l'Etoile, bioMérieux, France). If more than two species were isolated, the samples were defined as contaminated without further identification.

### Statistical analysis

All statistical analyses were performed using SPSS 27.0 (SPSS Inc., Chicago, IL, USA) and MedCalc (MedCalc Software, Ostend, Belgium) software. Normality was detected by the Kolmogorov–Smirnov test. Numeric variables were expressed as the mean ± standard deviation or median with interquartile ranges as appropriate. Categorical variables were presented as numbers or percentages and were analyzed using the Chi-square test. The UF-5000 BACT and WBC counts were compared with urine culture results using receiver operating characteristic (ROC) curve analysis. Sensitivity, specificity, negative predictive value, and positive predictive value were calculated to evaluate the optimal cut-off points. Cohen’s kappa consistency test was used to analyze the agreement between UF-5000 bacterial information flags and urine culture results. A *P*-value < 0.05 was considered statistically significant.

## RESULTS

### Characteristics of the urine samples

Among the 823 urine cultures, 540 (65.6%) were negative, and 283 (34.4%) were positive. Of the 283 positive samples, 31 were positive for yeast, and the remaining 252 (31.8%) were positive for bacteria. Among the 252 culture-positive specimens, the bacterial colony levels in 29 specimens (11.5%) were 10^4^ CFU/mL to 10^5^ CFU/mL, and those in 223 specimens (88.5%) were ≥10^5^ CFU/mL. A single species was identified in 244 of the positive samples, and two species were isolated in eight samples. *Escherichia coli*, *Klebsiella pneumoniae*, *Enterococcus faecium*, and *Enterococcus faecalis* were the most frequently isolated bacterial species. Other identified microorganisms are presented in Table 1.

**TABLE 1:** Microorganisms identified in culture-positive samples with ≥10^4^ CFU/mL bacterial counts^*a*^

Microorganism	n	%
*Escherichia coli*	113	44.84
*Klebsiella pneumoniae*	23	9.13
*Enterococcus faecium*	17	6.75
*Enterococcus faecalis*	13	5.16
*Klebsiella oxytoca*	9	3.57
*Proteus mirabilis*	8	3.17
*Pseudomonas aeruginosa*	7	2.78
*Streptococcus agalactiae*	6	2.38
Other Gram-negative bacteria	26	10.32
Other Gram-positive bacteria	22	8.73
Mixed culture	8	3.17

^*a*^ Mixed culture was defined as two species isolated from the culture samples.

### Diagnostic value of BACT and WBC counts in predicting UTIs

**Fig 1:**
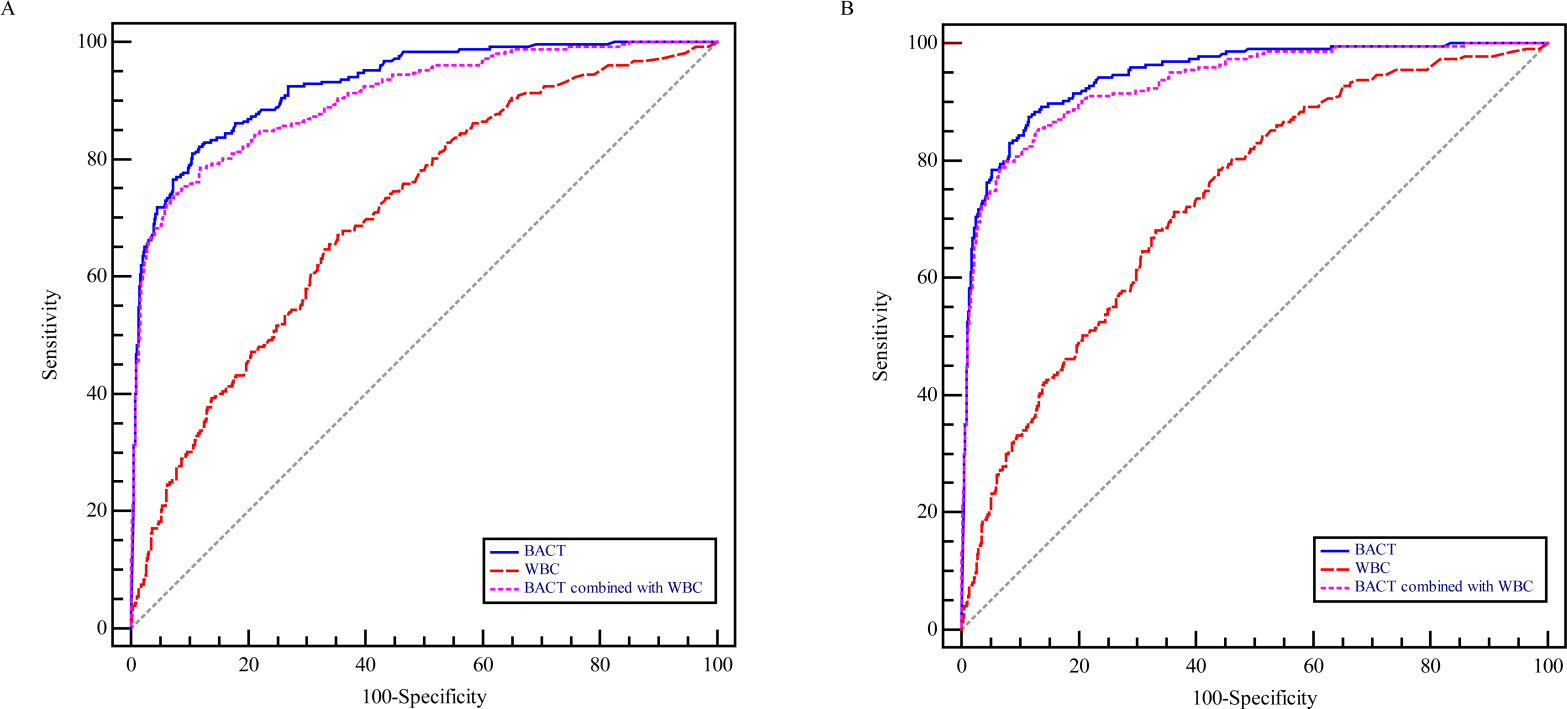
Comparison of ROC curves showing the performance of BACT, WBC count, and combined BACT and WBC count analysis and ROC curves obtained from urine culture results at ≥10^4^ CFU/mL (**A**) and ≥10^5^ CFU/mL (**B**) bacterial growth.

ROC curves were generated to explore the diagnostic value of BACT and WBC counts in the total population, as shown in Fig. 1. The areas under the curves (AUCs) achieved for the BACT counts were 0.927 (95% CI: 0.907 to 0.944) and 0.946 (95% CI: 0.928 to 0.961) at ≥10^4^ CFU/mL and ≥10^5^ CFU/mL bacterial growth, respectively, and the corresponding AUCs achieved for WBC counts were 0.708 (95% CI: 0.675–0.73) and 0.732 (95% CI: 0.699–0.762), respectively. We further assessed the combined diagnostic performance of WBC and BACT and found it was not superior to that of BACT analysis alone, with the corresponding AUCs being 0.905 (95% CI: 0.882–0.928) and 0.933 (95% CI: 0.914–0.952), respectively.

### Determining the best cut-off point to exclude negative urine culture samples

Then, we invested in the best cut-off point for bacteria levels to exclude patients with negative urine cultures and reduce unnecessary urine analysis. Sensitivity (SEN) and negative predictive value (NPV) were important indexes in discriminating culture-negative samples in the screening test. We excluded the higher SEN (97.5% and 99.0%) cut-off points for BACT count due to the increased number of false-positive samples and lower SPE. Therefore, the best BACT count cut-off points were 42.2/µL and 100.2/µL at ≥10^4^ CFU/mL and ≥10^5^ CFU/mL bacterial growth, respectively (Table 2).

**TABLE 2:** Diagnostic performance of UF-5000 versus urine culture results in the total population^*a*^^,^^*b*^

Variable SEN	BACT count (/μL)					
	80.0	85.0	90.0	95.0	97.5	99.0
Cutoff	440.8/1001.1	223.3/497.6	109.7/247.1	42.2/100.2	29.1/44.4	14.2/27.1
SPE	89.6/92.1	82.4/89.3	74.4/82.6	60.1/71.4	54.3/59.6	38.7/51.3
PPV	78.3/79.8	69.3/75.7	62.2/67.5	52.8/56.3	49.9/48.6	42.9/44.4
NPV	90.6/92.1	92.1/93.9	94.2/95.5	96.4/97.4	97.9/98.3	98.6/99.3
No. of TPs	202/178	214/190	227/201	240/212	246/217	249/221
No. of FNs	50/45	38/33	25/22	12/11	6/6	3/2
No. of TNs	484/524	445/508	402/470	325/406	293/339	209/292
No.of FPs	56/45	95/61	138/97	215/163	247/230	331/277

^*a*^ 252 positive cases with ≥10^4^ CFU/mL and 223 positive cases with ≥10^5^ CFU/mL bacterial growth.

^*b*^ BACT, bacteria; SEN, sensitivity; SPE, specificity; PPV, positive predictive value; NPV, negative predictive value; TP, true positive; FN, false negative; TN, true negative; FP, false positive.

### Agreement between the results of bacterial information flags and urine cultures

The comparison of the UF-5000 bacterial information flags and urine culture results is summarized in Table 3. We applied the ideal bacterial cut-off value (42.2/µL) to exclude urine culture-negative samples. Twenty-six (26/96, 27.1%) cases of Gram-positive microorganisms and 109 (109/125, 87.2%) cases of Gram-negative microorganisms were correctly discriminated by UF-5000. We also performed an analysis on the agreement between the UF-5000 bacterial information flags and urine culture results, obtaining a kappa value of 0.227 (*P* < 0.001).

**TABLE 3:** Consistency evaluation of UF-5000 BACT-Info flags versus urine culture results with a bacterial count ≥42.2/µL^*a*^

Flagging Sysmex UF-5000	Culture				
	Gram Pos	Gram Neg	Mixed culture	Negative	Total
Gram Pos	26	10	2	58	96
Gram Neg	2	109	0	14	125
Gram Pos/Neg	4	17	0	6	27
Unclassified	0	4	0	22	26
UTI	2	1	0	45	48
No flag	17	46	0	70	133
Total	51	187	2	215	455

^*a*^ Pos, Positive; Neg, Negative; No flag indicated the absence of bacterial information. If mixed culture bacteria included both Gram-negative microorganisms or Gram-positive microorganisms, we regarded them as Gram-negative or Gram-positive bacteria.

### Establishing an optimal angle for discriminating bacterial growth patterns

**Fig 2:**
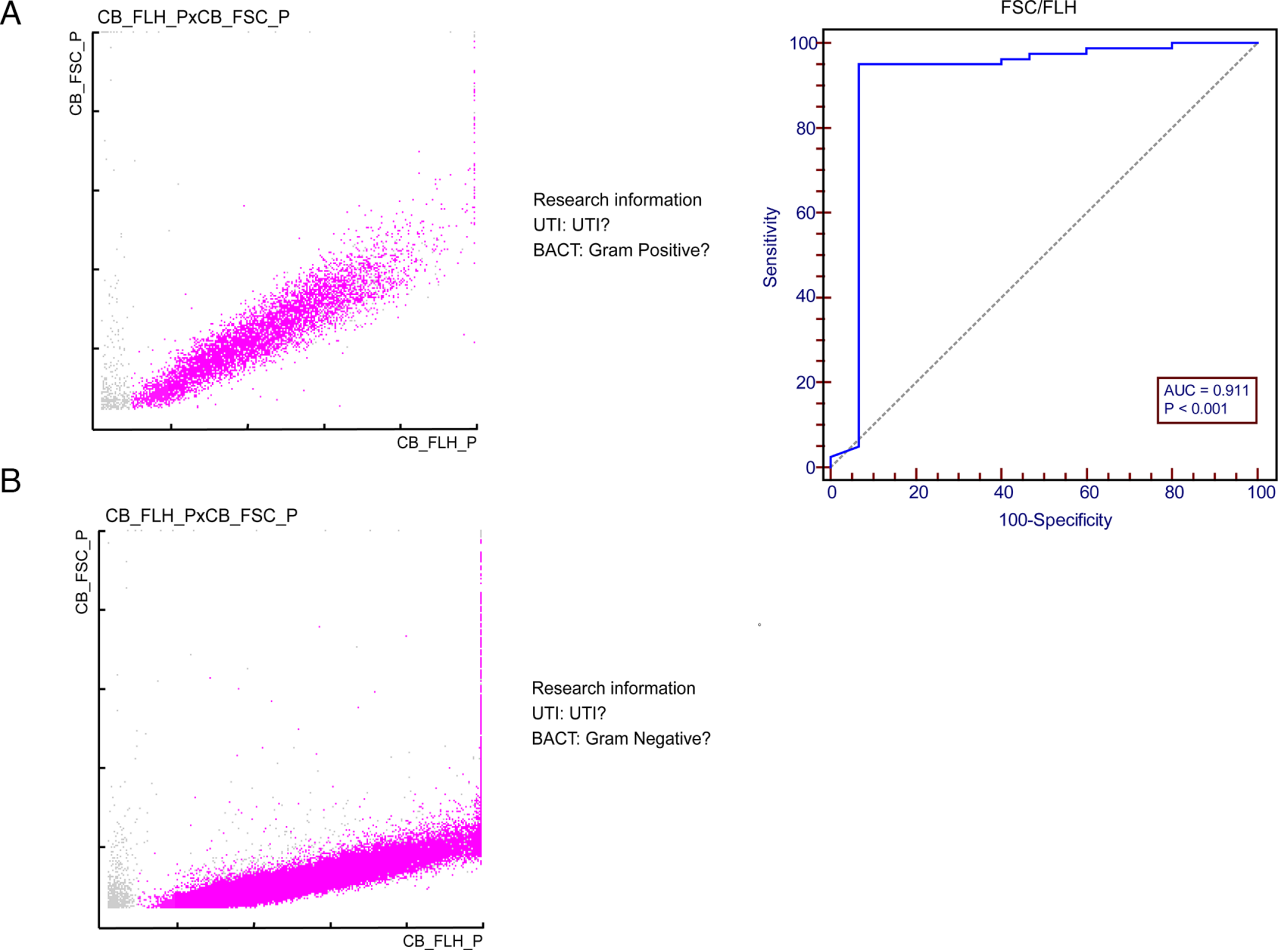
Flagging flow cytometry for bacteria was performed during UTI screening of samples with Gram-negative (**A**) and Gram-positive bacteria (**B**). Receiver operating characteristic curves were used to compare the ability of FSC/FLH to discriminate between Gram-negative and Gram-positive bacteria in samples with ≥10^4^ CFU/mL bacterial growth.

To explore the optimal angle for discriminating bacterial growth patterns, we assessed 550 samples and only enrolled positive urine culture samples without background interference. Finally, 80 Gram-negative samples and 15 Gram-positive samples were analyzed. The typical Gram-positive and Gram-negative scatter plots are shown below (Fig. 2). The median B_FSC parameter was significantly lower in Gram-negative than in Gram-positive (31.6 ch vs 59.8 ch, *P* < 0.001) samples, and median B_FLH parameter was higher in Gram-negative than in Gram-positive samples (101.2 ch vs 87.6 ch, *P* = 0.078). The median B_FSC/B_FLH ratio remarkedly differed in Gram-negative and Gram-positive samples (0.28 vs 0.63, *P* < 0.001) and B_FSC/B_FLH ratio optimal cut-off value was 0.55 for discriminating Gram-negative and Gram-positive bacteria, with a sensitivity of 95.0% and a specificity of 93.3% obtained from the ROC curve (Fig. 2). According to the relationship between slope and angle, the best angle from the X-axis was 28°.

### Verifying the ideal angle in the validation cohort

One hundred eighty urine specimens were culture-positive in the validation cohort at ≥10^4^ CFU/mL bacterial growth. We only enrolled urine specimens with a bacteria count ≥42.2/µL. Therefore, 176 specimens were eventually included in our analysis. Gram-negative bacteria were found in 80.7% and Gram-positive bacteria in 19.3% of the specimens. A single species was detected in 171 specimens, and two species were identified in five specimens. These five specimens each contained two Gram-negative bacteria. The angles of the scattergram distribution patterns of these specimens were examined and compared with the results of urine culture, which took an angle of 28° as the threshold angle. The results showed that most of the Gram-negative bacteria had a low-angle pattern (<28°) (Table 4).

**TABLE 4:** Specimens were classified according to the angle with a bacterial count ≥42.2/µL

	<28°	˃28°	Total
Gram-negative bacteria	127	15	142
Gram-positive bacteria	9	25	34
Total	136	40	176

## DISCUSSION

Considering the complexity of the disease in hospitalized patients and being able to perform comparisons with other studies, we used two criteria for positive urine culture. Urine cultures with bacterial growth of ≥10^4^ CFU/mL and ≥10^5^ CFU/mL were considered positive, respectively, and the corresponding favorable rates of culture were 31.8% (252/792) and 28.2% (223/792). The findings are similar to previously published data (14, 19). Gram-negative bacteria accounted for the majority of the bacteria identified by culture; *E. coli* was the most abundant Gram-negative bacterium. These culture results have also been validated in other studies (15, 20, 21).

It was worth noting that the diagnostic value of bacteria counts alone outperformed that of leukocyte counts combined with bacteria counts regardless of the level of bacterial growth. There is no consensus on whether the combined analysis of bacteria and leukocyte counts could improve the diagnostic value of UF-5000 (15, 16, 19, 22). Although the sensitivity and negative predictive value were slightly improved, the number of false-positive specimens was increased. Therefore, bacterial parameters alone were an essential indicator to compare with urine culture results.

Due to differences in the study design, criteria for positive urine cultures, and patient characteristics, there is no universally applicable cut-off point for bacterial levels in the Sysmex UF-5000 analysis. According to the literature, the critical value of bacterial levels ranged from 30/µL to 582.22/µL (14, 15, 19, 23). When ≥10^4^ CFU/mL bacterial growth level was considered as the standard for positive urine culture. Haugum et al. showed that the best cut-off point for bacteria levels in the general population was 30/µL, with a sensitivity of 95.2% and a specificity of 67.8% (15). Ren et al. reported that the best cut-off point for bacteria levels was 30/µL, with a sensitivity of 95.7% and a specificity of 76.8% (19). These results were comparable to ours. With ≥10^5^ CFU/mL bacterial growth level as the criterion for a positive specimen, the data from Millán-Lou et al. were similar to our findings. Their ideal cut-off point for bacteria levels was 89.4/µL, with a sensitivity of 94.8% and a specificity of 69.2% (22).

We explored the ability of the UF-5000 analyzer to identify bacteria in our laboratory. Overall, the agreement between the bacterial information flags and urine culture results was fair (Kappa = 0.227). Our data were in line with the findings of Christy et al. and Chen et al. (Kappa = 0.212, Kappa = 0.339, respectively) (23, 24). We further analyzed the reasons behind the lower kappa value, which included the poor ability of UF-5000 in differentiating Gram-positive bacteria and the UTI screening criteria set by the UF-5000 instrument (WBC ≥ 10/µL + BACT ≥ 100/µL), resulting in a high false-negative rate at the No flag group. The peptidoglycan layers of Gram-positive bacteria are thicker than those of Gram-negative bacteria, and the fluorescent dye does not easily pass through the cell wall, affecting nucleic acid staining (19). Therefore, the ability of UF-5000 to identify Gram-positive bacteria is weaker than that of Gram-negative bacteria; the screening strategy of leukocytes combined with bacteria in the analysis of UTI fails to detect specimens that only had higher bacterial values, leading to an increased false-negative rate. Based on the data in Table 4, we found that UF-5000 has an advantage in detecting Gram-negative bacteria, with a specificity of over 90% and a positive predictive value of over 80%. Several recent articles on predicting bacterial patterns with UF-5000 also confirmed that UF-5000 has advantages in distinguishing Gram-negative bacteria (25–27).

Our study assessed the value of B_FSC and B_FLH research parameters in identifying Gram-positive and Gram-negative bacteria with a UF-5000 urine analyzer. The median B_FSC parameters differed in the Gram-negative and Gram-positive bacteria (31.6 ch vs 59.8 ch, *P <* 0.001). Our results were comparable to those reported in previous studies. Rosa et al. and Gessoni et al. analyzed B_FSC and B_FLH parameters in their respective studies with a UF-1000i urine analyzer, and they found that median B_FSC parameters were significantly lower in Gram-negative than that in Gram-positive bacteria (22.1 ch vs 44.6 ch, *P <* 0.001; 20.6 ch vs 40.7 ch, *P <* 0.001) (17, 18). The B_FSC parameters reflect the size of formed components such as bacteria. These differences in B_FSC values could be explained by the fact that Gram-positive bacteria aggregate in irregular chains and/or irregular grape-like clusters, resulting in a larger and more variable size, while Gram-negative bacteria tend to stay in suspension as single cells (18, 28). The B_FLH parameters represent the fluorescence intensity of bacterial nucleic acids. The difference in B_FLH parameters between Gram-negative and Gram-positive bacteria may be related to the thicker peptidoglycan layer of Gram-positive bacteria, which leads to the lower permeability of fluorescent dyes (19). While another view showed different division patterns of Gram-positive and Gram-negative bacteria may affect the nucleic acid content of the bacteria (29). Rosa et al.’s study reported that the fluorescence signal intensity in Gram-positive bacteria is weaker than in Gram-negative bacteria (17). Therefore, we considered the B_FSC/B_FLH ratio could distinguish Gram-negative and Gram-positive bacteria. Compared our data with those of Yumi et al. (29), we found that these two angles were very similar (28° vs 30°). Furthermore, 699 urine samples were enrolled to validate the angle. Among the 136 urine samples that had the low angle pattern (<28°) in the validation cohort, 127 samples were confirmed to contain Gram-negative bacteria. Based on this finding, we could infer that an angle less than 28° indicated a Gram-negative infection; an angle greater than 28° suggested a Gram-positive infection.

In our study, we found that when ≥10^4^ CFU/mL and ≥10^5^ CFU/mL were considered as criteria for positive specimen in urine culture, the application of UF-5000 resulted in a reduction of bacterial culture by 42.6% (337/792) and 52.7% (417/792), respectively. Meanwhile, the false-negative rates were 3.6% (12/337) and 2.6% (11/417). Furthermore, we confirmed the diagnostic value of UF-5000 in identifying Gram-negative bacteria and established the best angle to distinguish Gram-negative and Gram-positive bacteria. However, there are some limitations in our research. First, the study was a single-center study that only included inpatients and outpatients who visited our hospital; second, the enrolled urine samples were not rigorously screened. For example, we did not assess the patients' electronic records to confirm whether the patients had been treated with antibiotics before specimen retention, nor did we exclude samples with mucus, excessive turbidity, or hematuria. Considering that patient populations may vary in different healthcare facilities, antibiotic treatment before urine specimen collection could affect bacteria shapes and the diversity of specimen types could affect the best cut-off of bacteria, which limits the generalizability of the findings. In the future, we will expand the sample size and set strict criteria for urine samples to conduct multi-center studies. Despite these limitations, the strength of our study relied on being the first study assessing the B_FSC and B_FLH research parameters with a UF-5000 urine analyzer and providing useful information about identifying Gram-negative bacteria.

### Research conclusion

In conclusion, we established an optimal bacterial cut-off point suitable for our laboratory with sufficient sensitivity and negative predictive value and reduced unnecessary urine culture. UF-5000 has a significant advantage in detecting Gram-negative bacteria. When the bacterial scatter plot angle is less than 28°, the possibility of Gram-negative bacterial infection is more than 80%.

## Data Availability

The data that support the findings of this study are available from the corresponding author, upon reasonable request.

## References

[1] Dubbs SB, Sommerkamp SK. 2019. Evaluation and management of urinary tract infection in the emergency department. Emerg Med Clin North Am 37:707–723. doi:10.1016/j.emc.2019.07.007 31563203

[2] Klein RD, Hultgren SJ. 2020. Urinary tract infections: microbial pathogenesis, host-pathogen interactions and new treatment strategies. Nat Rev Microbiol 18:211–226. doi:10.1038/s41579-020-0324-0 32071440 PMC7942789

[3] Geerlings SE. 2016. Clinical presentations and epidemiology of urinary tract infections. Microbiol Spectr 4. doi:10.1128/microbiolspec.UTI-0002-2012 27780014

[4] Shang Y-J, Wang Q-Q, Zhang J-R, Xu Y-L, Zhang W-W, Chen Y, Gu M-L, Hu Z-D, Deng A-M. 2013. Systematic review and meta-analysis of flow cytometry in urinary tract infection screening. Clin Chim Acta 424:90–95. doi:10.1016/j.cca.2013.05.014 23721948

[5] Mejuto P, Luengo M, Díaz-Gigante J. 2017. Automated flow cytometry: an alternative to urine culture in a routine clinical microbiology laboratory? Int J Microbiol 2017:8532736. doi:10.1155/2017/8532736 29090008 PMC5635286

[6] Yang SS-D, Yang C-C, Chen Y-S, Chang S-J. 2021. A performance comparison of the fully automated urine particle analyzer UF-5000 with UF-1000i and Gram staining in predicting bacterial growth patterns in women with uncomplicated urinary tract infections. BMC Urol 21:24. doi:10.1186/s12894-021-00791-x 33579236 PMC7881468

[7] Geerts N, Jansz AR, Boonen KJM, Wijn R, Koldewijn EL, Boer AK, Scharnhorst V. 2015. Urine flow cytometry can rule out urinary tract infection, but cannot identify bacterial morphologies correctly. Clin Chim Acta 448:86–90. doi:10.1016/j.cca.2015.06.020 26123581

[8] Íñigo M, Coello A, Fernández-Rivas G, Carrasco M, Marcó C, Fernández A, Casamajor T, Ausina V. 2016. Evaluation of the SediMax automated microscopy sediment analyzer and the Sysmex UF-1000i flow cytometer as screening tools to rule out negative urinary tract infections. Clin Chim Acta 456:31–35. doi:10.1016/j.cca.2016.02.016 26921459

[9] Wang J, Zhang Y, Xu D-W, Shao W-J, Lu Y. 2010. Evaluation of the Sysmex UF-1000i for the diagnosis of urinary tract infection. Am J Clin Pathol 133:577–582. doi:10.1309/AJCP1GT2JXOCQBCZ 20231611

[10] Herráez O, Asencio MA, Carranza R, Jarabo MM, Huertas M, Redondo O, Arias-Arias A, Jiménez-Álvarez S, Solís S, Zamarrón P, Illescas MS, Galán MA. 2018. Sysmex UF-1000i flow cytometer to screen urinary tract infections: the URISCAM multicentre study. Lett Appl Microbiol 66:175–181. doi:10.1111/lam.12832 29223137

[11] van der Zwet WC, Hessels J, Canbolat F, Deckers MML. 2010. Evaluation of the Sysmex UF-1000i urine flow cytometer in the diagnostic work-up of suspected urinary tract infection in a Dutch general hospital. Clin Chem Lab Med 48:1765–1771. doi:10.1515/CCLM.2010.342 20726812

[12] Broeren MAC, Bahçeci S, Vader HL, Arents NLA. 2011. Screening for urinary tract infection with the Sysmex UF-1000i urine flow cytometer. J Clin Microbiol 49:1025–1029. doi:10.1128/JCM.01669-10 21248088 PMC3067737

[13] Pieretti B, Brunati P, Pini B, Colzani C, Congedo P, Rocchi M, Terramocci R. 2010. Diagnosis of bacteriuria and leukocyturia by automated flow cytometry compared with urine culture. J Clin Microbiol 48:3990–3996. doi:10.1128/JCM.00975-10 20739491 PMC3020858

[14] Enko D, Stelzer I, Böckl M, Schnedl WJ, Meinitzer A, Herrmann M, Tötsch M, Gehrer M. 2020. Comparison of the reliability of Gram-negative and Gram-positive flags of the Sysmex UF-5000 with manual Gram stain and urine culture results. Clin Chem Lab Med 59:619–624. doi:10.1515/cclm-2020-1263 33068381

[15] Haugum K, Haugan MS, Skage J, Tetik M, Jakovljev A, Nilsen H-J, Afset JE. 2021. Use of Sysmex UF-5000 flow cytometry in rapid diagnosis of urinary tract infection and the importance of validating carryover rates against bacterial count cut-off. J Med Microbiol 70:001472. doi:10.1099/jmm.0.001472 34898416 PMC8744275

[16] Alenkaer LK, Pedersen L, Szecsi PB, Bjerrum PJ. 2021. Evaluation of the sysmex UF-5000 fluorescence flow cytometer as a screening platform for ruling out urinary tract infections in elderly patients presenting at the emergency department. Scand J Clin Lab Invest 81:379–384. doi:10.1080/00365513.2021.1929441 34237238

[17] De Rosa R, Grosso S, Bruschetta G, Avolio M, Stano P, Modolo ML, Camporese A. 2010. Evaluation of the Sysmex UF1000i flow cytometer for ruling out bacterial urinary tract infection. Clin Chim Acta 411:1137–1142. doi:10.1016/j.cca.2010.03.027 20359474

[18] Gessoni G, Saccani G, Valverde S, Manoni F, Caputo M. 2015. Does flow cytometry have a role in preliminary differentiation between urinary tract infections sustained by gram positive and gram negative bacteria? An Italian polycentric study. Clin Chim Acta 440:152–156. doi:10.1016/j.cca.2014.11.022 25433140

[19] Ren C-Y, Wu J-B, Jin M-C, Wang X, Cao H-C. 2018. Rapidly discriminating culture-negative urine specimens from patients with suspected urinary tract infections by UF-5000. Bioanalysis 10:1833–1840. doi:10.4155/bio-2018-0175 30295053

[20] Kim H, Kim HR, Kim TH, Lee MK. 2019. Age-specific cutoffs of the Sysmex UF-1000i automated urine analyzer for rapid screening of urinary tract infections in outpatients. Ann Lab Med 39:322–326. doi:10.3343/alm.2019.39.3.322 30623625 PMC6340846

[21] Ippoliti R, Allievi I, Rocchetti A. 2020. UF-5000 flow cytometer: a new technology to support microbiologists' interpretation of suspected urinary tract infections. Microbiologyopen 9:e987. doi:10.1002/mbo3.987 31908145 PMC7066453

[22] Millán-Lou MI, García-Lechuz JM, Ruiz-Andrés MA, López C, Aldea MJ, Revillo MJ, Rezusta A. 2018. Validation and search of the ideal cut-off of the Sysmex UF-1000i flow cytometer for the diagnosis of urinary tract infection in a tertiary hospital in Spain. Front Med (Lausanne) 5:92. doi:10.3389/fmed.2018.00092 29686988 PMC5900046

[23] Christy P, Sidjabat HE, Lumban Toruan AA, Moses EJ, Mohd Yussof N, Puspitasari Y, Fuadi MR, Marpaung FR. 2022. Comparison of laboratory diagnosis of urinary tract infections based on leukocyte and bacterial parameters using standardized microscopic and flow cytometry methods. Int J Nephrol 2022:1–8. doi:10.1155/2022/9555121 PMC9167024 35669495

[24] Chen Y-B, Zhang Z-S, Diao Y-J, Wang W-N, Zhu Y, Li J-M, Wang G-Q, Zhao Y, Lin Z-Z, Wu Y-B, Jing J. 2023. Combination of UC-3500 and UF-5000 as a quick and effective method to exclude bacterial urinary tract infection. J Infect Chemother 29:667–672. doi:10.1016/j.jiac.2023.03.008 36921761

[25] Wang H, Han F-F, Wen J-X, Yan Z, Han Y-Q, Hu Z-D, Zheng W-Q. 2023. Accuracy of the Sysmex UF-5000 analyzer for urinary tract infection screening and pathogen classification. PLoS One 18:e0281118. doi:10.1371/journal.pone.0281118 36724192 PMC9891513

[26] Kim SH, Kim GR, Kim EY, Song SA, Yang J, Shin JH. 2022. Clinical usefulness of BACT count and BACT-info flag of UF-5000 for screening for urinary tract infection and prediction of gram-negative bacteria. Clin Lab 68. doi:10.7754/Clin.Lab.2022.220217 36546738

[27] De Rosa R, Grosso S, Lorenzi G, Bruschetta G, Camporese A. 2018. Evaluation of the new Sysmex UF-5000 fluorescence flow cytometry analyser for ruling out bacterial urinary tract infection and for prediction of Gram negative bacteria in urine cultures. Clin Chim Acta 484:171–178. doi:10.1016/j.cca.2018.05.047 29803898

[28] Jiménez-Guerra G, Heras-Cañas V, Valera-Arcas MD, Rodríguez-Grangér J, Navarro JM, Gutiérrez-Fernández J. 2017. Comparison between urine culture profile and morphology classification using fluorescence parameters of the Sysmex UF-1000i urine flow cytometer. J Appl Microbiol 122:473–480. doi:10.1111/jam.13354 27860075

[29] Asutake YY, Iguchi MH, Da SO. 2013. Comparisons of the back scattergram pattern by fully utomated integrated urine analyzer UX-2000 and microscopic examination results using gram stain. Sysmex J Int 23

